# A Human Protein Interaction Network Shows Conservation of Aging Processes between Human and Invertebrate Species

**DOI:** 10.1371/journal.pgen.1000414

**Published:** 2009-03-13

**Authors:** Russell Bell, Alan Hubbard, Rakesh Chettier, Di Chen, John P. Miller, Pankaj Kapahi, Mark Tarnopolsky, Sudhir Sahasrabuhde, Simon Melov, Robert E. Hughes

**Affiliations:** 1 Prolexys Pharmaceuticals, Salt Lake City, Utah, United States of America; 2 School of Public Health, University of California Berkeley, Berkeley, California, United States of America; 3 Buck Institute for Age Research, Novato, California, United States of America; 4 McMaster University Medical Center, Hamilton, Ontario, Canada; Stanford University Medical Center, United States of America

## Abstract

We have mapped a protein interaction network of human homologs of proteins that modify longevity in invertebrate species. This network is derived from a proteome-scale human protein interaction Core Network generated through unbiased high-throughput yeast two-hybrid searches. The longevity network is composed of 175 human homologs of proteins known to confer increased longevity through loss of function in yeast, nematode, or fly, and 2,163 additional human proteins that interact with these homologs. Overall, the network consists of 3,271 binary interactions among 2,338 unique proteins. A comparison of the average node degree of the human longevity homologs with random sets of proteins in the Core Network indicates that human homologs of longevity proteins are highly connected hubs with a mean node degree of 18.8 partners. Shortest path length analysis shows that proteins in this network are significantly more connected than would be expected by chance. To examine the relationship of this network to human aging phenotypes, we compared the genes encoding longevity network proteins to genes known to be changed transcriptionally during aging in human muscle. In the case of both the longevity protein homologs and their interactors, we observed enrichments for differentially expressed genes in the network. To determine whether homologs of human longevity interacting proteins can modulate life span in invertebrates, homologs of 18 human FRAP1 interacting proteins showing significant changes in human aging muscle were tested for effects on nematode life span using RNAi. Of 18 genes tested, 33% extended life span when knocked-down in *Caenorhabditis elegans*. These observations indicate that a broad class of longevity genes identified in invertebrate models of aging have relevance to human aging. They also indicate that the longevity protein interaction network presented here is enriched for novel conserved longevity proteins.

## Author Summary

Studies of longevity in model organisms such as baker's yeast, roundworm, and fruit fly have clearly demonstrated that a diverse array of genetic mutations can result in increased life span. In fact, large-scale genetic screens have identified hundreds of genes that when mutated, knocked down, or deleted will significantly enhance longevity in these organisms. Despite great progress in understanding genetic and genomic determinants of life span in model organisms, the general relevance of invertebrate longevity genes to human aging and longevity has yet to be fully established. In this study, we show that human homologs of invertebrate longevity genes change in their expression levels during aging in human tissue. We also show that human genes encoding proteins that interact with human longevity homolog proteins are also changed in expression during human aging. These observations taken together indicate that the broad patterns underlying genetic control of life span in invertebrates is highly relevant to human aging and longevity. We also present a collection of novel candidate genes and proteins that may influence human life span.

## Introduction

Genetic modulation of life span is ultimately mediated through proteins, and the mechanisms that allow this control must necessarily involve the interaction of multiple proteins. As a biological pathway, aging is a pleiotropic process, and many of the proteins identified as influencing this process have a proportionate pleiotropy of function. Modulations of the levels in a single protein have been found that provide robust increases in life-span for an organism [1],[2], but contributions from many genes are expected to dictate longevity in all organisms. This idea is supported by an investigation of yeast protein-protein interaction networks that found that proteins related to aging have a significantly higher connectivity than expected by chance [3]. Similarly, a second group found that their computational model suggested aging genes have more connections in interaction networks, and that this may be useful in identifying new aging genes [4]. Therefore, a useful way to identify novel genes with roles that affect life span is to identify their gene product's interactions with known aging-associated proteins.

A role for protein interactions in processes is most apparent at the level of protein complexes that assemble to carry out a particular function. Likewise, protein interactions that mediate signaling cascades demonstrate how interactions functionally translate into a biological pathway. Indeed, biological processes are built of hierarchical protein-protein interaction assemblies that together carry out the overall physiological process. Therefore, the identification of interactions that a protein participates in can be an informative way to pursue an understanding of the protein's function. A common method for identifying protein interactions is the yeast two-hybrid system (Y2H), which uses the interaction of two proteins to reconstitute a transcription factor that then activates expression of a reporter gene [5]. An important development in the Y2H approach was the introduction of the screening of libraries of potential interacting proteins [6]. This development made it possible to identify novel protein interactions. Novel interactions impart a suggested role in a physiological process for proteins based on the established involvement of their interaction partner in that process.

Recently, high throughput approaches have expanded this idea to a systems-scale level: investigators can identify the network of interactions that occur among a large set of proteins, and from this infer the relationships of those proteins in, as well as their contribution to, the system. Such an approach has been used to interrogate the protein interaction networks that underlie model organisms [6]–[12], human cells [13],[14], and organisms responsible for infectious diseases [15]–[17]. Biological processes such as vulval development in nematodes [18], and familial neurodegenerative diseases [19]–[21] have also been the subject of large-scale Y2H interaction mapping. From these studies, many hypotheses for new participants in biological pathways have emerged.

The results from high-throughput protein interaction studies are known to contain false-positive (i.e. biologically irrelevant) interactions intermingled with the biologically relevant interactions. Independent large-scale studies of the same system may not necessarily distinguish the two [22], although detection of an interaction in more than one study is strong evidence for the authenticity of the interaction. An additional approach to address interaction validity is to use features of the network itself to provide evidence for the physiological relevance of the identified interactions. Protein interaction networks behave as scale-free networks, and the resultant properties such as path length and clustering features can be mined with bioinformatic methods to evaluate the properties of a given interaction within the network [23],[24]. Comparisons with other phenotypic data can provide further support. An observation of similar regulation using gene expression analysis has been used to establish confidence in protein interactions by a number of groups [8],[11],[15],[25],[26]. Shared gene ontology annotations [27] can also be used to identify characteristics of proteins that support the link(s) suggested by the interactions [15].

## Results

### Interaction Network of Human Homologs of Invertebrate Longevity Proteins

**Figure 1:**
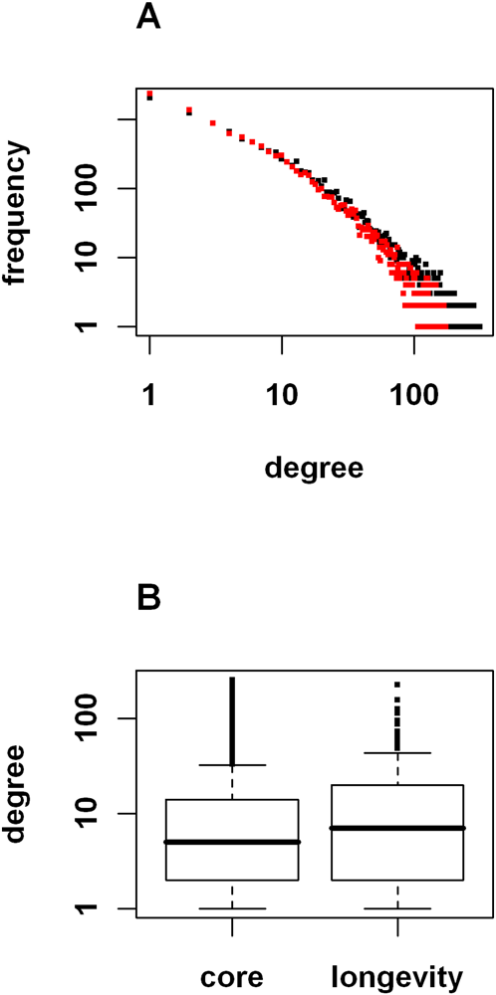
Node Degree Distributions in Core Network and Longevity Network. Panel A shows the node degree distribution in unfiltered and Core protein interaction networks. A log-log plot of node degrees in both unfiltered and Core interaction networks appears as a straight line indicating that both are scale free. Black circles represent node degrees of 11,280 proteins in a network of 114,698 interactions. Red circles show the node degree distribution after removal of bait proteins with >87 interactions and prey proteins with >231 interactions. The Core Network contains 70,358 binary interactions among 10,425 unique proteins. Panel B shows the node degree distributions of the Core and longevity networks represented as box plots. The average node degree in the Core Network is 13.5. The average node degree for the 175 longevity proteins is 18.8. Median node degrees (indicated by thick horizontal lines) for the core and longevity networks are respectively 5.0 and 7.0.

We performed a comprehensive survey of the published literature on the genetics of aging as studied in model systems (yeast, fly, nematode and mouse) and identified 363 genes that have been reported to increase life span when mutated. Most of these genes were curated in the SAGE KE Genes/Interventions Database (http://sageke.sciencemag.org). The remainder were culled from published large-scale genetic screens for longevity phenotypes [28]–[32]. In order to characterize these longevity genes/proteins in the context of a human protein interaction network we sought to analyze their protein interaction partners in a large human protein interaction database. We have used high-throughput yeast two-hybrid methods to construct a large network comprised of 114,689 unique binary interactions between fragments of human proteins. This network was generated using results from ∼345,000 individual yeast two hybrid screens. Aspects of the Prolexys human protein interaction network and methods used to generate it have been described previously [15],[21],[33]. The 114,689 interaction network was filtered to create a Core Network with 70,358 unique binary interactions between protein fragments representing 10,425 unique genes curated as NCBI RefSeq entries. The Core Network was generated by removal of “sticky” proteins identified using a K-means clustering method [15]. Exclusion of bait proteins with >87 interactions and prey proteins with >231 interactions resulted in removal of 44,331 interactions and 855 nodes (i.e. unique genes) from the unfiltered network. Figure 1A shows a log-log graph of node degree distributions of the unfiltered network (black circles) and the Core Network (red circles). The fact that the degree distribution appears as a straight line on a logarithmic plot indicates that the Core Network is scale-free [23],[34]. This Core Network was queried to determine the interaction properties of human protein homologous to proteins experimentally implicated regulation of life span. A masked version of the complete Core Network is shown in Table S6.

The majority of genes and proteins identified as having a role in modulation of life span were discovered in yeast, fly and nematode. We therefore identified the human orthologs and homologs of these invertebrate longevity genes according to definitions used in NCBI's Homologene (http://www.ncbi.nlm.nih.gov/entrez/query.fcgi?DBhomologene) and the Karolinska Institute's Inparanoid Database (http://inparanoid.cgb.ki.se/). Of the 363 invertebrate longevity genes identified, 252 have human homologs and 175 of these homologs are represented in the Core Network of the Prolexys protein interaction database (Table S1). The proteins encoded by the 175 human homologs of invertebrate longevity genes were observed to interact with 2,163 additional human proteins in the yeast two-hybrid assays. This longevity protein interaction network ultimately consists of a total of 3,271 binary connections between the 2,338 proteins (Table S2). When the longevity network was derived from the Core Network it was immediately apparent that the longevity homologs were unusually highly connected with an average node degree of 18.8 and a median node degree of 7.0 (see Table S1 for individual node degrees). These values are notably higher that average and median node degrees of 13.5 and 5.0 observed for the entire Core Network (Table S6). Figure 1B shows a box plot comparing the distribution of node degrees for the 175 human longevity protein homologs and the Core Network from which the longevity sub-graph was derived. This indicates that human homologs of longevity proteins comprise a group of highly connected hubs in the Core Network. The increase in the median node degree for the longevity proteins indicates that this distribution is not due to the effect of outliers.

**Figure 2:**
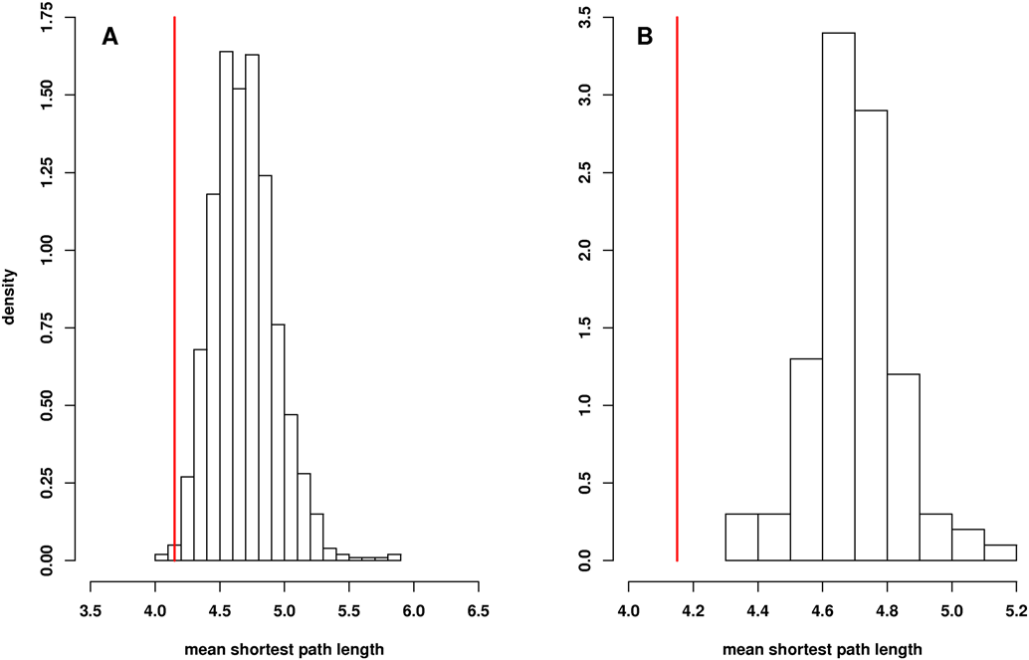
Path length analysis of longevity genes in Core Network. Panel A shows a comparison of the mean shortest path length of the 175 genes in the longevity cohort to the average shortest path length distribution in the Core Network. The histogram shows the distribution of mean shortest path lengths observed in 1,000 sets of 175 genes randomly selected from the 10,430 unique genes present as nodes in the Core Network. The mean shortest path length for all genes is 4.61. By comparison, the mean shortest path length for the 175 longevity genes is 4.15 (vertical red line). The p-value for the significance of this difference is 0.004. Panel B shows path length analysis for interactions among longevity homologs using randomized networks. The mean shortest path length between the 175 longevity protein homologs in the network is 4.15 (vertical red line). The distribution of mean shortest path lengths between these proteins in 100 networks with randomly assigned connections is shown. The peak of the distribution in the randomized networks is 4.73. As none of the values from the permutation distribution was less than 4.15, the p-value for the significance of this difference is <0.01.

A path length analysis was performed to determine whether the network of longevity protein homologs were more closely connected to each other than would be expected by chance. Figure 2A shows the average mean shortest path length in 1,000 sets of 175 proteins selected at random from the Core Network is 4.61 as compared to 4.15 for the longevity network (p = 0.004). This result is consistent the prediction that proteins with shared functions (in this case the modification of life span) are more likely to be closely associated in the network than would be expected by chance. To determine whether this path length difference is a trivial result of the high average node degree in the longevity network, we performed a path length analysis using networks with randomized connections. In order to do this, the edges in the Core Network of 70,358 binary interactions were randomly reassigned while preserving the node degree of each individual protein. The average path length between the longevity protein homologs present in 100 randomized core interactomes was then determined. As shown in Figure 2B, we found that the average shortest distance between any two longevity proteins (4.15) is significantly less than the average distance of 4.73 (±0.13) between these proteins in the 100 networks with randomly assigned interactions (p<0.01). This result shows that the decreased path length observed in the longevity network is not simply a feature of high node degrees but is in fact dependent on the connections between the specific interacting proteins included in the longevity network.

### Expression of Genes Encoding Human Longevity Homologs and Their Interacting Proteins during Human Aging

The 2,163 human proteins that interact with the invertebrate longevity homologs are not known to be involved directly in aging or longevity phenotypes. However, because of their ability to bind directly to known longevity proteins in the yeast two-hybrid assay, these can be considered as candidate longevity proteins. To validate potential roles for the interacting proteins in human longevity we looked for evidence that the expression of genes encoding these proteins might be changed during the aging process. To do this, we compared the network to DNA microarray datasets comparing gene expression in human skeletal muscle from cohorts of young and old healthy volunteers [35]. In this microarray study, skeletal (*vastus lateralis*) muscle biopsies from healthy older and younger adult men and women were compared using gene expression profiling. After quantile normalization, the number of genes significantly differentially expressed with age was determined by performing, on a probe-by-probe basis, 24,354 two-sample t-tests. To control the family-wise error rate (FWER), the significant genes were chosen at 5% using Holm's step-down method. FWER was used to insure a low probability of any false positives among this list. Using a false discovery rate cut-off of 5%, a large number of genes were found to be differentially expressed as a consequence of age [35].

To integrate the longevity interactome with the gene expression data, we asked whether any of the genes encoding longevity proteins or their interactors (“1° interactors”) were significantly changed in the transcript profiles from old vs. young human cohorts. Of the 175 longevity proteins, 169 were represented on the microarray used in this study by 210 probes. We determined how many of the 210 probes had a significant association of expression and age using analyses based on loess normalized intensities converted to log scale. HOPACH (Hierarchical Ordered Partitioning and Collapsing Hybrid) was then used to cluster the resulting genes and generate plots of similarly expressed genes. This analysis identified 54 of the 210 probes (52 of 169 unique genes) as being differentially expressed between the old and young cohorts (FDR q-value<0.05). The differentially expressed aging gene homologs are listed in Table 1.

**Table 1:** Human Longevity Gene Homologs with Significant Expression Changes in Muscle.

Human Homolog	Geometric Mean Ratio (old/young)	FDR Adjusted p-value	Model Organism Gene Name	Model Organism
**amino acid metabolism**				
AMT	1.1	3.80E-02	GCV1	*S. cerevisiae*
GCN1L1	1.1	2.10E-02	GCN1	*S. cerevisiae*
MSRA	0.9	2.10E-02	Eip71CD	*D. melanogaster*
**axonal guidance**				
FEZ1	1.2	9.60E-05	unc-76	*C. elegans*
FEZ2	1.8	1.20E-08	unc-76	*C. elegans*
**cell adhesion**				
ILK	1.2	9.60E-05	pat-4	*C. elegans*
PARVB	0.8	2.10E-02	pat-6	*C. elegans*
**cell cycle**				
CDK8	0.9	3.70E-02	SSN3	*S. cerevisiae*
HSMPP8	1.2	4.09E-05	T09A5.8	*C. elegans*
**cell signaling**				
DUSP10	1.2	2.00E-04	puc	*D. melanogaster*
FRAP1	0.9	2.00E-03	let-363	*C. elegans*
GNAI1	0.9	5.90E-03	GPA2	*S. cerevisiae*
OPA1	0.9	4.60E-03	eat-3	*C. elegans*
PRKAG1	0.7	2.40E-07	SNF4	*S. cerevisiae*
PRKAG2	0.8	1.60E-02	SNF4	*S. cerevisiae*
RAB10	1.3	1.50E-04	rab-10	*C. elegans*
RRAGD	0.7	1.60E-06	GTR2	*S. cerevisiae*
SGKL	1.4	7.30E-05	sgk-1	*C. elegans*
**cytoskeletal processes**				
CNN3	1.2	1.40E-02	SCP1	*S. cerevisiae*
DNCH2	1.1	2.30E-02	che-3	*C. elegans*
FLNB	1.3	5.50E-04	cher	*D. melanogaster*
TAGLN	1.4	2.60E-03	SCP1	*S. cerevisiae*
**energy metabolism**				
ACO2	0.9	1.60E-02	aco-2	*C. elegans*
AOX1	1.2	4.70E-03	F55B11.1	*C. elegans*
ATP5F1	0.9	1.00E-02	asb-2	*C. elegans*
ATP6V0A1	1.3	5.90E-05	unc-32	*C. elegans*
COX4I1	0.9	1.50E-04	W09C5.8	*C. elegans*
COX5B	0.9	2.90E-03	cco-1	*C. elegans*
CYC1	0.9	3.60E-03	cyc-1	*C. elegans*
IDH1	0.8	7.00E-08	F59B8.2	*C. elegans*
IDH3A	0.6	2.80E-05	F43G9.1	*C. elegans*
UGP2	0.9	2.60E-03	K08E3.5	*C. elegans*
UQCRFS1	0.8	1.20E-08	isp-1	*C. elegans*
**mRNA maturation**				
NUP98	1.1	1.40E-02	NUP100	*S. cerevisiae*
RBPMS	1.2	2.00E-02	mec-8	*C. elegans*
**protein catabolism**				
EDEM1	1.2	9.80E-05	MNL1	*S. cerevisiae*
SH3MD2	0.9	2.60E-03	POSH	*D. melanogaster*
SH3RF2	1.6	5.80E-04	POSH	*D. melanogaster*
UCHL5	0.9	4.10E-02	ubh-4	*C. elegans*
**response to stress**				
HSPA9B	0.9	1.70E-02	hsp-6	*C. elegans*
**transcription**				
RFX1	1.2	3.70E-05	daf-19	*C. elegans*
RFX3	0.9	1.10E-02	daf-19	*C. elegans*
**translation**				
EEF1A1	1.3	9.70E-05	Ef1alpha48D	*D. melanogaster*
EEF1A2	1.2	1.50E-04	Ef1alpha48D	*D. melanogaster*
MRPL47	0.8	2.60E-03	B0261.4	*C. elegans*
**transport of molecules**				
ABCC5	1.2	1.80E-03	mrp-5	*C. elegans*
FABP3	0.6	3.50E-07	lbp-7	*C. elegans*
FLJ10074 (SCYL2)	0.8	9.17E-05	SCY1	*S. cerevisiae*
SLC25A3	0.9	2.60E-03	C33F10.12	*C. elegans*
STX1A	1.1	2.00E-02	unc-64	*C. elegans*
**unknown**				
KIAA0931 (PHLPPL)	1.2	1.16E-04	CYR1	*S. cerevisiae*
LIM (PDLIM5)	1.5	9.96E-08	eat-1	*C. elegans*

**Figure 3:**
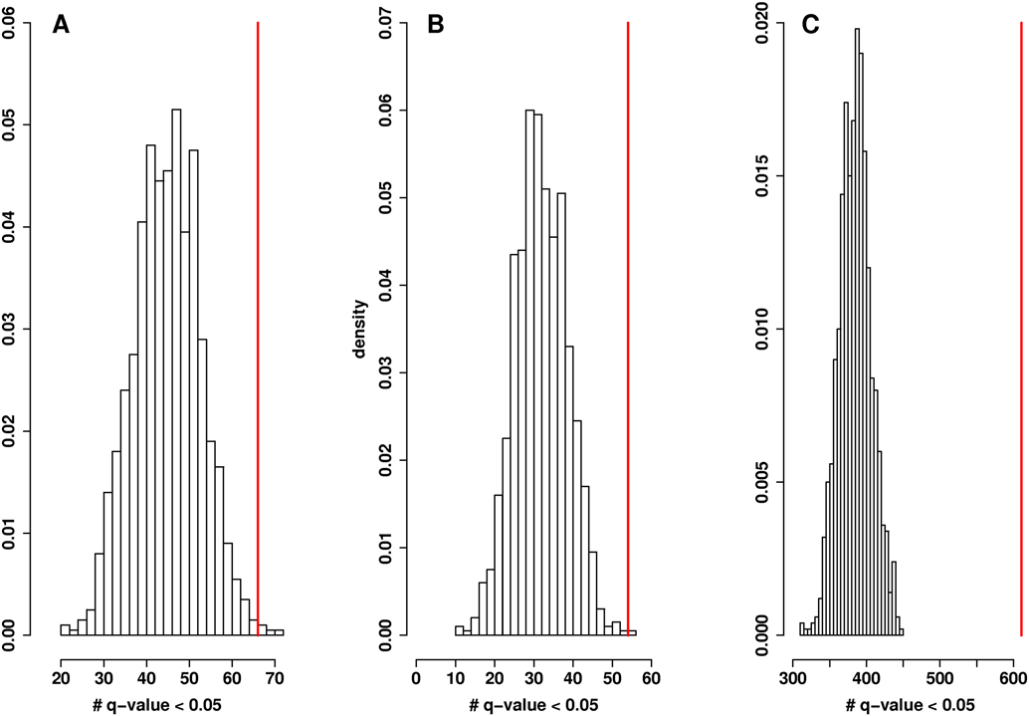
Significance of gene expression changes for longevity gene homologs and interacting proteins. The permutation distributions (based on 1,000 permutations of the array label) for the number of significant probes (based on FDR value in the association of age versus expression) for three different sets: A. human homologs of aging genes (based on 1,000 random draws of 291 probes), B. longevity gene homologs present in the interaction network (based on 1,000 random draws of 210 probes), and C. 1° interactor protein genes (based on 1,000 random draws of 2,507 probes). Vertical red lines indicate values (number of probes with FDR-based q-value<0.05) for the original experimental datasets from which the p-values of these three tests are derived.

To see whether this was unusual, we included an additional test to determine whether this set of probes is more enriched in genes associated with age than one would expect by pure chance. We drew randomly from the original list of genes probes (24,354 probes genes) 210 at a time and for each of these random draws, examined the number of genes probes significantly associated with age at the same level of significance. However, among only 1 of the 1,000 random draws we performed, did that many or more significant genes probes come up, implying a significant enrichment among this set (p-value = 0.001; see Figure 3B). A permutation test for all 236 gene longevity gene homologs present on the microarray (represented by 291 probes) is shown in Figure 3A. We found among the longevity gene homologs (regardless of whether they were present in the interaction network data), 66 out of 291 probes were significantly associated with age. However, among only 4 of the 1000 random draws we performed, did that many significant genes come up, implying a significant enrichment among this set as well (p = 0.004).

We next evaluated the 2,507 probes that correspond to genes encoding 2,036 of the 2,163 1° interactors in the longevity interactome network. We repeated the analyses described above for the longevity proteins. Among the 1° interactors, 611 out of the 2,507 probes (581 of 2,036 genes) were significantly associated with age. In 1,000 random draws of 2,507 probes, none contained 611 (or more) significant genes, demonstrating significant enrichment among the set of 1° interactors (p<0.001; see Figure 3C).

These statistical analyses clearly demonstrate that genes encoding human homologs of invertebrate longevity genes and genes encoding their interacting proteins are highly enriched among genes with a statistically significant change in expression between young and old muscle tissue in human. This result is somewhat surprising in that these genes are derived primarily from experiments done in invertebrate models, and thus one might not expect a priori to see age-dependent changes in expression levels in human tissue. Two preliminary conclusions are suggested by these observations: 1) longevity genes discovered in invertebrate models are likely to play some roles in human longevity and 2) cells and tissues appear to modulate expression levels of such longevity genes during the aging process in human. A list of human homologs of invertebrate aging genes and genes encoding interacting proteins that show significant expression changes in aging human muscle are shown in Table S3.

**Figure 4:**
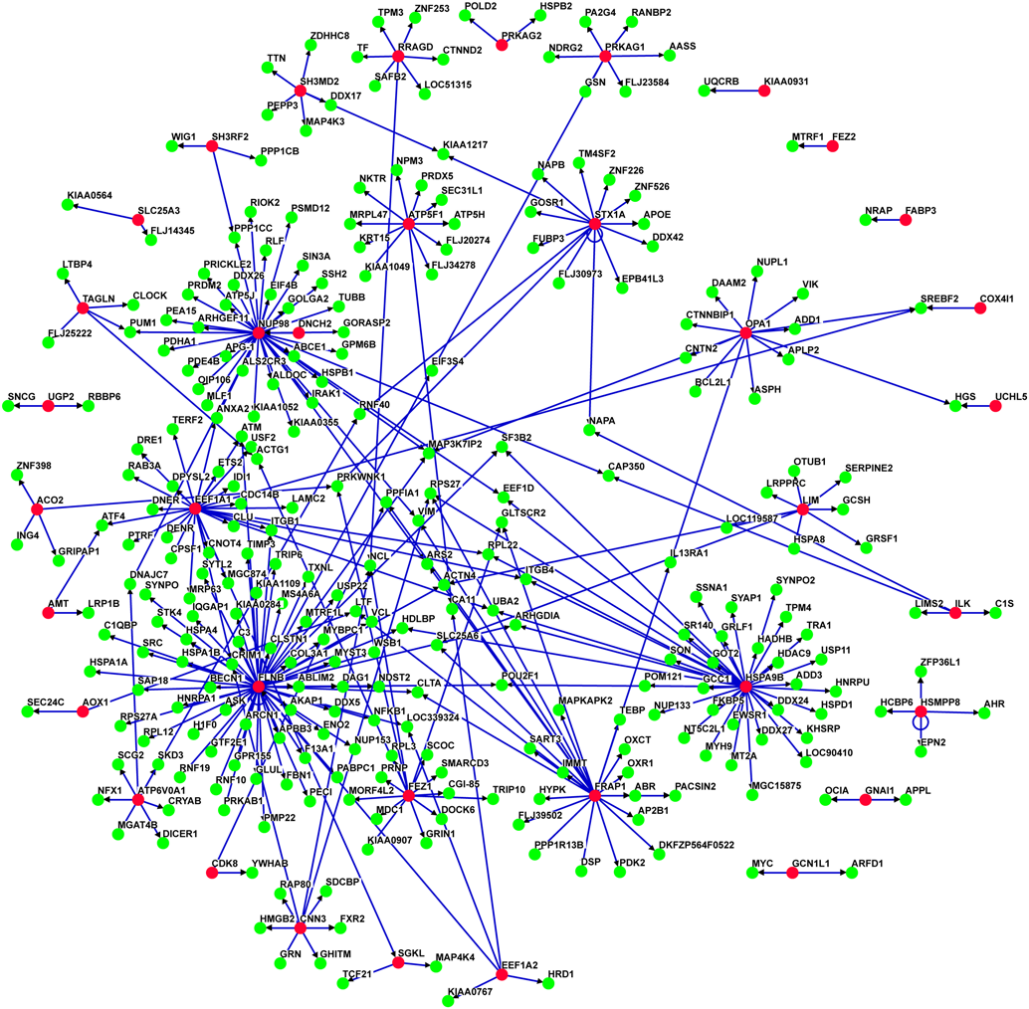
Subset of Longevity Network including only those genes whose expression is significantly changed in young vs old human muscle. Longevity gene homologs are shown in red; interacting proteins are shown in green. The network contains 339 interactions among 325 proteins.

Figure 4 shows a subnetwork of the longevity interactome. This subnetwork includes only those genes whose expression is significantly changed in the aging microarray data. This subnetwork contains 339 interactions among 325 proteins, roughly 10% of the interactions in the larger network. We consider proteins in this network to be of high interest for further studies. An example of one group of interest is FRAP1 (mTOR) and its interacting proteins. FRAP1 has total of 63 interacting protein interactions in the longevity network.

FRAP1 has a well-established role in longevity, with loss of function mutations in the FRAP1 orthologs in both nematodes [36] and yeast [30],[32] leading to increased life span. Our results suggest that FRAP1 may also have a role in human longevity. Human FRAP1 interacts with 63 proteins that have not previously been shown to be involved in longevity. Some of these have functions that are consistent with known FRAP1 functions of FRAP1, e.g. an interaction with RPS27, a component of the small ribosomal subunit may be related to the function of FRAP1 in translational control; similarly, nuclear import of FRAP1 is necessary for signaling through S6K and an interaction with TPR supports the idea that mTOR associates with the nuclear pore [37]. Interestingly, mRNA levels for 24 of the 63 partners (38%) of FRAP1 are significantly different between young and old patient samples. Proteins that can interact with FRAP1 are thus frequently expressed differentially with age in human. FRAP1 interacting proteins that show significant changes in gene expression during aging in human muscle are shown in Table 2.

**Table 2:** Human FRAP1 Interacting Proteins with Significant Expression Changes in Muscle.

FRAP1 Interacting Protein Name	Geometric Mean Ratio (old/young)	FDR Adjusted p-value
**cell adhesion**		
DSP	0.9	3.12E-02
PPFIA1	1.2	2.58E-03
**cell signaling**		
ABR	1.3	7.17E-04
GLTSCR2	1.1	2.92E-02
IL13RA1	1.1	1.49E-02
MAPKAPK2	0.9	1.98E-02
PACSIN2	1.2	7.47E-03
PPP1R13B	1.1	1.08E-02
**cytoskeletal processes**		
VIM	1.5	2.92E-05
**energy metabolism**		
IMMT	0.7	2.32E-06
OXCT	1.3	1.41E-02
PDK2	0.8	1.65E-02
**mRNA maturation**		
SART3	1.3	8.04E-05
**response to stress**		
ARS2	1.2	3.22E-04
OXR1	0.9	2.87E-02
TEBP (PTGES3)	1.2	1.25E-02
**translation**		
RPS27	1.2	1.78E-04
**transport of molecules**		
AP2B1	0.8	1.67E-05
CLTA	1.2	5.27E-05
LTF	1.1	3.26E-02
SLC25A6	1.2	2.19E-03
**unknown**		
DKFZP564F0522	0.8	3.50E-03
FLJ39502	1.1	4.98E-02
HYPK	1.1	2.14E-02

**Figure 5:**
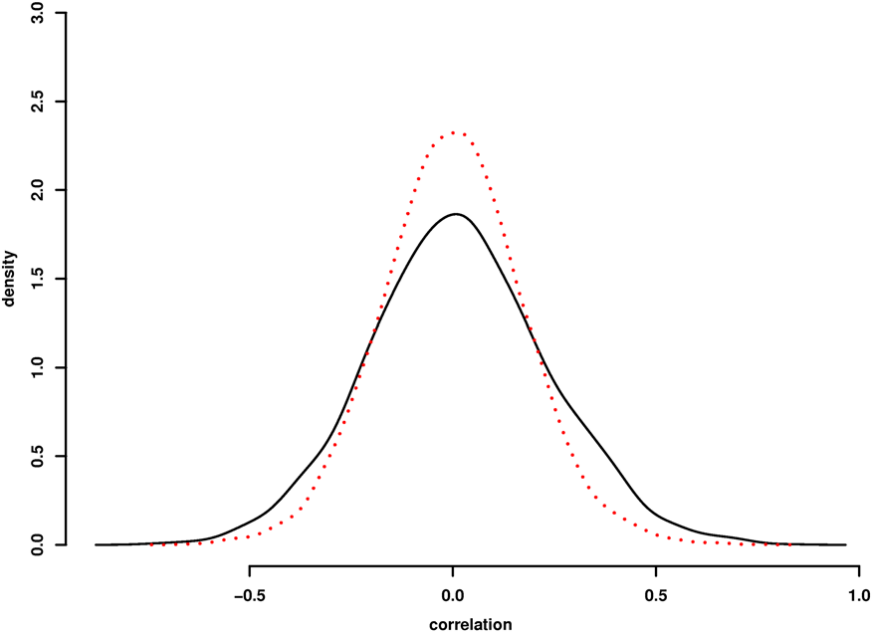
Correlation of gene expression changes with binary protein interactions. Distribution of transcriptional expression correlations for binary protein interaction pairs in the longevity network is shown in black. Distributions of correlation for randomized binary pairs is shown in red. The experimental network shows enrichment for both positively and negatively correlated binary pairs. Approximate inference via Two-sample Kolmogorov-Smirnov test confirms significant differences in the two distributions of correlations (p<0.00001).

To determine whether there is a relationship between protein interaction and a correlation in gene expression between protein pairs in this network, we compared the distribution of both negative and positive gene expression correlations with binary interactions. Figure 5 shows the distribution of gene expression correlations for the experimentally derived longevity network as compared to simulated networks of genes with randomly assigned binary connections. Both positively and negatively co-regulated protein pairs are enriched in the longevity interaction network relative to that observed in randomized networks. This observation supports the idea that interacting proteins are transcriptionally co-regulated [38]. A list of the binary pairs with significant age-dependent transcriptional co-regulation is shown in Table S4.

### Validating Human FRAP1 Interacting Proteins in a *C. elegans* Life Span Assay

**Figure 6:**
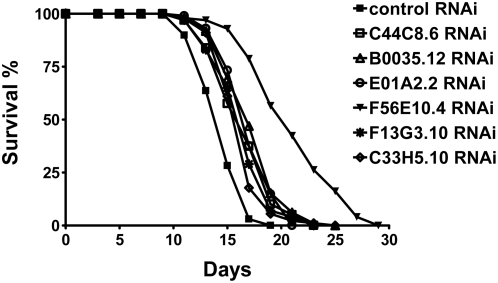
Kaplan–Meier survival curves for *C. elegans* treated with RNAi knock-down of genes encoding homologs of six human FRAP1 interacting proteins. Human homologs corresponding to nematode genes are as follows: MAPKAP2 (C44C8.6); SART3 (B0035.12); ARS2 (E01A2.2); RPS27 (F56E10.4); HYPK (F13G3.10); DKFZP564F0522 (C33H5.10).

In order to test the hypothesis that interacting partners of human longevity homologs might themselves be longevity proteins we tested a group of these for effects on life span in *C. elegans*. The 24 FRAP1 interacting proteins with significant gene expression changes in aging human muscle are listed in Table 2. Of these 18 were tested for their ability to modulate life span in *C elegans* using RNAi mediated knock-down (six of 24 were not tested because reagents were not available in our RNAi library). Wild-type N2 *C. elegans* were fed *E. coli* expressing double-stranded RNA corresponding to genes encoding 18 FRAP1 interacting proteins and life spans were determined in two independent experiments. Of the 18 genes tested in this way, six reproducibly extended the life span of *C. elegans* by >10% (Figure 6). These genes are listed in Table 3. The gene showing the greatest effect on life span after RNAi treatment is RPS27. Knock-down of rps-27 expression in nematode resulted in 50% and 44% increases in life span in two independent experiments. Mammalian RPS27 encodes a zinc finger-containing protein component of the 40S ribosomal subunit [39]. Several studies have established that TOR signaling can modulate life span in yeast [30],[32] and fly [40]. It has been demonstrated further that inhibition of translation can also extend life span indicating that loss-of-function in TOR signaling modulates aging through an effect on rates of translation [41]–[43]. Since RPS27 is a component of the ribosome and interacts with FRAP1 (Tor), it is likely that the life span extension seen in the rps-27 knock-down is due to an effect on rates of translation either through TOR signaling, direct effects on ribosome structure, or a combination of the two.

**Table 3:** Effect of Knock-down of FRAP1 Interacting Proteins on *C. elegans* Life Span.

Gene	Functional group	*C. elegans* homolog	RNAi clone	Life span (days)		Extension a	*p* b	n c
				Mean	Max			
			control	13.1, 14.7	18, 19			134, 99
MAPKAPK2	cell signaling	C44C8.6	IV-1G22	14.7, 16.8	22, 23	12%, 14%	0.0003, <0.0001	120, 99
SART3	mRNA maturation	B0035.12	IV-6E07	14.7, 17.6	22, 25	12%, 20%	0.0001, <0.0001	120, 98
ARS2	response to stress	E01A2.2	I-1F02	14.6, 17.4	22, 21	11%, 18%	0.0002, <0.0001	134, 101
RPS27	translation	F56E10.4	V-1C03	19.7, 21.2	30, 29	50%, 44%	<0.0001, <0.0001	108, 99
HYPK	unknown	F13G3.10	I-3J23	14.6, 16.7	22, 23	11%, 14%	0.0003, <0.0001	136, 100
DKFZP564F0522	unknown	C33H5.10	IV-3P02	15.5, 16.5	22, 25	18%, 12%	<0.0001, <0.0001	117, 90

a mean life span extension compared to animals treated with control RNAi.

b *p* values were calculated using the log-rank method.

c numbers of animals scored.

The fact that 33% of the candidates tested had a significant effect on life span extension is noteworthy. Previous genome wide screens in *C. elegans* using RNAi have reported that less than 1% of the nematode genome may encode genes that can extend life span when knocked-down [28],[29].

## Discussion

We present here a large protein interaction network comprised of human homologs of genes known to influence longevity in invertebrate systems and their interacting proteins. To compile this list of homologs, we selected genes that confer increased life span when mutated, deleted or knocked down in yeast, flies or nematodes. The longevity homolog sub-network (3,271 interactions) is derived from a much larger Core Network (70,358 interactions) that was generated in an unbiased fashion using a random high throughput yeast two hybrid process. The Core Network was generated from larger network after removal of sticky proteins with very high node degrees [15],[21],[33]. Analysis of the human longevity interactome presented here show that the 175 human longevity homologs are more closely connected that would be expected by chance, with a mean path length of 4.15 as compared to and average of 4.61 in the Core Network. Another striking feature of human homologs of invertebrate longevity proteins is their exceptionally high average node degree of 18.8 (as compared to an average of 13.5 in the Core Network). This observation indicates that human longevity protein homologs may function as hub proteins in the human interactome [44],[45]. The fact that longevity proteins are hubs may be indicative of their having a central role in cellular function. They may also function as nodes that connect and/or integrate functionally diverse cellular components and systems. It is interesting to consider the possibility that knock-down of these longevity genes may extend life span through a mechanism that involves uncoupling connections between cellular components of diverse function.

A striking conclusion of this study is dramatic degree of enrichment for genes encoding network proteins among genes that are transcriptionally modulated during aging in human muscle tissue. This correlation indicates that the network is enriched for proteins involved in human aging. This conclusion is consistent with the observation that human proteins interacting with the longevity homolog FRAP1 can increase life span when knocked-down in *C.elegans*. Overall these results provide evidence that the broad class of longevity proteins identified in invertebrates have a conserved role in processes of human aging and longevity.

## Materials and Methods

### Bait and Prey Construction

Complementary DNA was generated from poly(A)+ RNA isolated from multiple human tissues (including adult brain, fetal brain and liver) and inserted between the Gal4 transcriptional activation domain and the *Schizosaccharomyces pombe* URA4 coding region of pOAD.102 (prey plasmid) or the Gal4 DNA-binding domain and the *S. cerevisiae* MET2 coding region of pOBD.111 (bait plasmid). Yeast transformed with bait or prey plasmids were plated on medium lacking uracil (prey) or methionine (bait) to select for transformants expressing the markers fused to the cDNA inserts. Additional information about the plasmids, yeast strains and library construction can be found in Supplementary Information.

### Automated Yeast Two-Hybrid Screening Process

The two-hybrid expression plasmids, pOBD.111 and pOAD.102 used in this study have been described [15]. pOBD.111 and pOAD.102 are modifications of pOBD and pOAD [46]. The bait and prey yeast strains used were respectively, R2HMet (MATα ura3-52 ade2-101 trp1-901 leu2-3, his3-200 met2Δ::hisG gal4Δ gal80Δ) and BK100 (MATa ura3-52 ade2-101 trp1-901 leu2-3,112 his3-200 gal4Δ gal80Δ GAL2-ADE2 LYS2::GAL1-HIS3 met2::GAL7-lacZ), a derivative of PJ69-4A [47]. Bait and prey cDNA libraries were made using poly(A)^+^ RNA prepared from human tissues (see Table S5) by random primed cDNA synthesis followed by the PCR addition of yeast recombination tails. Both bait and prey cDNAs are cloned as a double fusion between the two-hybrid domain on the 5′ end of the insert and an ORF-selection marker on the 3′ end. Specifically, bait cDNA inserts were cloned between the GAL4 DNA binding domain and the TRP1 or MET2 coding regions, and prey inserts between the GAL4 transcriptional activation domain and URA3 [15]. These cDNAs were then cloned into linearized expression vectors by recombination in yeast [46]. Yeast transformed with bait were plated on medium lacking tryptophan or methionine to select for in-frame TRP1 or MET2 fusions, respectively, and prey were selected without uracil for in-frame URA3 fusions.

Y2H screens were performed in 96-well plates by mating in each well 5×10^6^ cells of a yeast clone expressing a single bait with 5×10^6^ clonally diverse cells from a prey library. After mating overnight, the Matings were plated using a Genesis Workstation 150 liquid handling robot (Tecan) onto medium that selected simultaneously for the mating event, the expression of the ORF-selection markers, and the activity of the metabolic reporter genes, ADE2 and HIS3. Yeast that grew on this selection medium (“positives”) were counted and transferred into liquid medium in a 96-well format using a MegaPix colony picking robot (Genetix). A maximum of 48 colonies per mating were picked. Searches that yielded more than 200 positives (∼2% of all searches) were considered to result from bait plasmids that activated transcription in the absence of specific protein-protein interactions, and were not analyzed further. Cloned inserts were amplified from plasmid PCR. Liquid cultures grown from positive yeast colonies were used as templates in PCR reactions that amplified either both bait and prey cDNA inserts, or prey inserts only in screens in which the baits had been sequenced before the matings. The PCR reactions were assembled in 384-well format using the Genesis Workstation 150 or a custom built (Zymark) PCR workstation that included a SciClone ALH 500 liquid handling robot (Zymark). PCR amplification took place in Primus-HT thermocyclers (MWG Biotech). The amplicons served as templates in DNA sequencing reactions. Identities of insert fragments were established by querying against the NCBI RefSeq database. The Y2H protein-protein interaction database is the result of two distinct workflow modes referred to as random and directed. In the random mode individual bait clones are picked randomly from a library and mated with a library of prey cDNAs. Directed searches, on the other hand, are matings of prey libraries with a single intentionally constructed bait cDNA clone whose identity is known *a priori*. In random searches, moreover, the identity of the bait is discovered – depending, again, on a particular workflow – either before or after the mating has been performed. The alternatives are to sequence both the bait and prey from Y2H positives (called positive-derived sequence) or to sequence the bait plasmid before mating (called pre-sequencing) requiring only the prey to be sequenced from positive diploids. All Y2H search data and DNA sequences used to determine interaction pairs reported in this study are included in Table S5.

### Homology Searches

A total of 363 genes that had been reported to increase life span when mutated yeast, fly, nematode and mouse species were compiled from SAGE KE and the published literature. We then screened for their respective clusters in Homologene and Inparanoid databases. The human genes among those clusters were deemed to be the orthologs of the respective invertebrate genes. Any additional human paralogs were also taken into consideration. The 363 invertebrate genes have homology to genes had human ortholog/paralog which resulted in a total of 252 human genes.

### Data Filtering

k-means clustering (k = 2) was applied sequentially to prey and baits in the core protein interaction database to define two populations of genes based on their number of partners [15]. Those interactions involving genes (i.e. baits with >87 interactions and preys with >231 interactions) were deemed promiscuous by this analysis and removed from the final dataset. The remaining interactions were referred to as the “Core Network”. The unfiltered core interactome had a total of 120,779 interactions involving 11,327 genes curated as NCBI Gene entries. The Core Network after filtering comprised of 71,814 interactions from 10,430 genes. The aging interactome reported here includes only interactions from the Core Network.

### Network Topology Analysis

To establish the basis for suitable null hypotheses, the process of deriving subnetworks from the large interaction network was performed 1000 times with sets of 175 genes randomly selected from one of two sources: 1) any gene contained in the Y2H PPI database or 2) genes in either Homologene or InParanoid having homologs of *C. elegans*, *D. melanogaster* or *S. cerevisiae*. Because the latter set corresponds to genes conserved from phylogenetically distant organisms it is referred to as “ancient.” In each iteration of the process, the 175 genes were used to query the Y2H PPI database and create subnetworks in a manner otherwise identical to that of the procedure for longevity homologs.

The mean shortest path length between any two aging genes in the actual longevity network was calculated. We simulated the Core Network 100 times, by rewiring the edges, preserving the node degree of each protein. The aging related human genes were then screened through 100 randomized networks, to generate 100 simulated longevity networks. We then calculated the mean shortest path length between any two aging genes in the 100 randomized networks. A one sided t-test was used to compare mean shortest path lengths of the experimentally derived data to those of 100 randomizations.

### Gene Expression Data Analysis

No background correction was performed given the very low levels of background intensity, however we performed loess normalization [48] on the entire set of probes to account for differences in the distribution of intensities among arrays. To select the genes that are differentially expressed with regards to age among the probes that matched our set of longevity proteins we performed, gene by gene, simple two-sample t-tests and used the Benjamini-Hochberg procedure [49] to derive adjusted q-values for the list of genes ranked by statistical significance. After deriving the number of significantly differentially expressed genes (based on an FDR cut-off of 5%), we wished to determine if this set of probes was significantly enriched with genes whose expression changes related to age, which motivated a permutation test to find whether the identification of a gene is related to life span extension was independent of differential expression with regards to the microarray data on muscle tissue in old and young subjects. We simply performed a large number of permutations on the longevity protein label for the total set of probes, each permutation randomly designated genes as either longevity protein genes or not and then among this random set, we performed the same procedure to find the number of significantly differentially expressed genes. After 1000 permutations, we have 1000 randomly generated numbers of significantly differentially expressed genes and we can compare our observed number to this null distribution to find the p-value of the test that these genes (related to life extension) or unrelated to age in human muscle. We performed an identical analysis for the 1° interactor genes.

### Correlation Analysis

To examine whether probes for genes encoding binary interaction pairs had more evidence of co-regulation in the microarray data, we examined correlation of log2 expression of probes of pairs of genes that were 1) connected directly and randomly chosen equal number of pairs of probes for pairs of genes unconnected in the network from the total list of probes on the Illumina array. For genes connected in the interactome represented by more than one probe, the correlation of all relevant pairs of probes were estimated (i.e., if there were 3 probes in one gene matched with 2 probes in another, this generated a total of 6 correlations). The purpose of this was to determine whether genes connected in the interactome were more related in expression than randomly drawn pairs of genes.

### *C. elegans* Life Span Assays

Animals were grown on NGM agar plates seeded with OP50 *E. coli* at 20°C. RNAi bacteria strains were cultured as previously described [50]. Wild-type N2 animals at the late L4 larval stage were fed with *E. coli* expressing different double-stranded RNAs and incubated at 25°C for life span experiments. 5-fluorodeoxyuridine (0.05 mg/ml) was added onto plates during the reproductive phase to eliminate progeny. Animals were transferred onto fresh plates every 3–6 days. The first day of adulthood is Day 1 in survival curves. Animals were scored as alive, dead or lost every other day. Animals that did not move in response to touching were scored as dead. Animals that died from causes other than aging, such as sticking to the plate walls, internal hatching or bursting in the vulval region, were scored as lost. In all life span assays, *E. coli* carrying the empty RNAi vector L4440 was fed to animals as controls. Statistical analyses were performed using the Prism 4 software (Graphpad Software, Inc., San Diego, CA, USA). Kaplan–Meier survival curves were plotted for each life span experiment and p values were calculated using the log-rank test [50].

## Supporting Information

Table S1 Invertebrate longevity genes and human homologs.
(0.11 MB XLS)

Table S2 Longevity Interaction Network.
(0.20 MB XLS)

Table S3 Significant expression changes in genes encoding Longevity Network proteins.
(0.19 MB XLS)

Table S4 Correlation in expression changes for genes encoding binary interactors.
(0.41 MB XLS)

Table S5 Yeast 2-hybrid screening data.
(11.69 MB XLS)

Table S6 Coded Core Network.
(1.00 MB CSV)
